# Social inclusion of families of children with Zika syndrome in light of Betty Neuman

**DOI:** 10.1590/1980-220X-REEUSP-2024-0214en

**Published:** 2025-01-31

**Authors:** Laís Helena de Souza Soares Lima, Maria Wanderléya de Lavor Coriolano-Marinus, Monique de Freitas Gonçalves Lima, Eliane Maria Ribeiro de Vasconcelos, Cleide Maria Pontes, Francisca Márcia Pereira Linhares

**Affiliations:** 1 Universidade Federal de Pernambuco, Programa de Pós-Graduação de Enfermagem, Recife, Pernambuco, Brazil.

**Keywords:** Family, Social Inclusion, Zika Virus Infection, Nursing Theory, Nursing, Familia, Inclusión Social, Infección por el Virus Zika, Teoría de Enfermería, Enfermería, Família, Inclusão Social, Infecção por Zika vírus, Teoria de Enfermagem, Enfermagem

## Abstract

**Objective:**

To identify the stressors and reactions related to the knowledge and experiences of families of children with congenital Zika virus syndrome on social inclusion in light of Betty Neuman.

**Method:**

Qualitative research, carried out in a hospital institution with nine mothers of children with Congenital Zika Syndrome. The data were collected between November and December 2020, from semi-structured interviews, and analyzed with the help of the IRAMUTEQ software and the manifest content analysis framework.

**Results:**

The analyses revealed three categories: Activities outside the home, highlighting the daily challenges faced outside the home, such as the lack of support in the educational system; Difficulties in adaptation, which highlighted physical and social barriers, such as the inadequacy of public spaces and the lack of social empathy; Knowledge about rights and inclusion, showing the lack of knowledge and uncertainty of families about their rights and social inclusion.

**Final considerations:**

The experiences and knowledge revealed the need for social interaction, adaptation, facing prejudice, seeking validity of rights and leading role in the context of social inclusion of these families.

## RESUMO

**Objetivo:**

Identificar os estressores e as reações relacionadas aos saberes e vivências das famílias de crianças com Síndrome Congênita do Zika vírus sobre inclusão social à luz de Betty Neuman.

**Método:**

Pesquisa qualitativa, realizada em instituição hospitalar com nove mães de crianças com Síndrome Congênita do Zika. Os dados foram coletados entre novembro e dezembro de 2020, a partir de entrevista semiestruturada, e analisados com auxílio do software IRAMUTEQ e o referencial da análise de conteúdo manifesto.

**Resultados:**

As análises revelaram três categorias: Atividades externas ao lar, destacando os desafios diários enfrentados fora de casa, como a falta de apoio no sistema educacional; Dificuldades de adaptação, que evidenciaram barreiras físicas e sociais, como a inadequação de espaços públicos e a falta de empatia social; Saberes sobre direitos e inclusão, mostrando o desconhecimento e a incerteza das famílias sobre seus direitos e a inclusão social.

**Considerações finais:**

As vivências e os saberes revelaram a necessidade de interação social, adaptação, enfrentamento de preconceito, busca pela validação de direitos e protagonismo no contexto da inclusão social dessas famílias.

## RESUMEN

**Objetivo:**

Identificar los estresores y reacciones relacionados con los conocimientos y experiencias de familias de niños con Síndrome Congénito del Virus Zika sobre la inclusión social a la luz de Betty Neuman.

**Método:**

Investigación cualitativa, realizada en una institución hospitalaria con nueve madres de niños con Síndrome Congénito de Zika. Los datos fueron recolectados entre noviembre y diciembre de 2020, a partir de una entrevista semiestructurada, y analizados con ayuda del software IRAMUTEQ y el marco de análisis de contenido manifiesto.

**Resultados:**

Los análisis revelaron tres categorías: Actividades fuera del hogar, destacando los desafíos cotidianos que se enfrentan fuera del hogar, como la falta de apoyo en el sistema educativo; Dificultades de adaptación, que pusieron de relieve barreras físicas y sociales, como la insuficiencia de los espacios públicos y la falta de empatía social; Conocimiento sobre derechos e inclusión, mostrando el desconocimiento y la incertidumbre de las familias sobre sus derechos e inclusión social.

**Consideraciones finales:**

Experiencias y conocimientos revelaron la necesidad de interacción social, adaptación, enfrentamiento de prejuicios, buscando validar derechos y protagonismo en el contexto de inclusión social de estas familias.

## INTRODUCTION

Congenital Zika Virus Syndrome (CZVS) emerged in Brazil in the last months of 2015 and is defined as a series of alterations found in newborns infected with the Zika virus (ZIKV) during the gestational period^(1,2)^. The context in which families of children with CZVS find themselves, due to the severity of children’s health condition and economic instability, characterized by changes in survival methods, active search for resources in health services and the ineffectiveness of government support, highlights individual, family and social vulnerability^(3,4)^. The presence of CZVS in the family setting has caused a great social and emotional impact due to the action of stressors that point to different patterns of behavioral functioning and social and family adaptation^(5)^.

The care and support network for this segment of the population needs to be increasingly cohesive. The greatest challenge lies in formulating public policies that guarantee families of children with CZVS the promotion of health and quality of life, as well as social assistance, full access to multidisciplinary health services, in an attempt to reduce barriers, strengthen access and eliminate inequalities^(3,6)^.

Social inclusion is defined as a set of actions that promote access to guarantees of life in society, such as health, education, employment, and the realization of rights for people who, for some reason – in this case, disability – find themselves in a disadvantaged relationship with the social system^(7)^. A potential approach to the realization of social inclusion is through support programs that offer innovative and sustainable ways to promote the skills and knowledge of parents and, thus, the health of their children with special needs as part of a broader strategy. Working from the perspective of social inclusion with health promotion means considering the concept of vulnerability by addressing deeper causes and poor health behaviors^(6)^.

Families of children with CZVS are in a vulnerable state, predominantly belonging to less privileged social strata and often forced to give up their professional occupations to dedicate themselves fully to the care of their children. Moreover, children with CZVS are subject to multiple forms of vulnerability – individual, social and institutional –, due to severe limitations in health conditions, low socioeconomic status and increasing dependence on health services, resulting from their physical and intellectual disabilities^(4−6)^.

Considering the above, the line of care proposed by Betty Neuman contributes to the development of actions that encourage the use of social and emotional support strategies with a view to strengthening the family system and its defense lines. Only with a strengthened system can relationships and interactions with the environment begin to generate positive effects, which contributes to balance and well-being^(8,9)^. When nursing care for families is guided by the line of thought based on Betty Neuman’s theory, from a holistic and comprehensive perspective, the construction of nurses’ care plan can promote balance in the constant interaction with stressors, through care focused on health promotion, thus contributing to strengthening these defense lines^(10)^.

This article aimed to identify the stressors and reactions related to the knowledge and experiences of families of children with CZVS on social inclusion in light of Betty Neuman.

## METHOD

### Study Design

This is a qualitative study, based on Betty Neuman’s Systems Model Theory, which approaches stressors as stimuli that can be destabilizing, of three natures: intrapersonal, which occurs within the limits of individuals’ own system; interpersonal, which occurs outside the limits of the system, from one individual to another; and extrapersonal, which also occurs outside the limits of the system, but at a greater distance from the system. This theory also demonstrates that the system is surrounded by lines of protection against the action of stressors that, when reached, generate reactions to the stressors^(8,11)^. The study was conducted and structured with reference to the COnsolidated criteria for REporting Qualitative research (COREQ)^(12)^.

### Study Setting

The study was carried out at a hospital institution in the municipality of Vitória de Santo Antão, Pernambuco, Brazil, which, among the services provided to the community, has the Multidisciplinary Assistance Center for Child Neurodevelopment (In Portuguese, *Núcleo de Assistência Multidisciplinar ao Neurodesenvolvimento Infantil* - NAMNI), a therapeutic reference for the Brazilian Health System (In Portuguese, *Sistema Único de Saúde* - SUS) in the state of Pernambuco in relation to the multidisciplinary approach for habilitation/rehabilitation of children with neurodevelopmental disorder.

### Data Source

Study participants consisted of mothers of children who provided direct care to children with CZVS, totaling nine women. Selection was made by convenience or voluntary sampling^(11)^, with all the mothers of children who were being monitored at NAMNI who met the following criteria: being a family member, over 18 years of age, who provides direct informal care to children with CZVS. All the mothers of children were eligible and agreed to participate in the interview.

### Data Collection and Organization

Data collection took place between November 2019 and February 2020 through semi-structured interviews, using questions for socioeconomic characterization, with the purpose of collecting general information to characterize the family member who provides and children who receive care, and questions that sought to identify experiences and knowledge related to social inclusion, as well as stressors, elements that strengthen families’ defense lines and patterns of coping with situations related to social inclusion, based on the assumptions of Betty Neuman’s theory (What activities does the family carry out with children?; What places/social environments do you frequent with your child? (alternatives); About the places you frequent: how do you feel/perceive regarding the participation of your family and your child in this place?; What do you like most and least about this place?; About the places you do not frequent: why do you not frequent them?; What rights do you consider important for your family and children?; How would you like to be helped in the participation of your children in the places you frequent?; For you, what is social inclusion?). As a way of improving the way the interviews were conducted, before applying the instrument to the participating mothers, a pilot test was carried out with two mothers of children with CZVS who were not being monitored at the research site. The interviews were conducted by the main author and held in a private room at NAMNI, with only the researcher and the interviewee present, individually, in order to guarantee the confidentiality and privacy of participants, with an average duration of approximately 30 minutes. The data were recorded with the aid of a digital recorder and, later, were transcribed in full.

### Data Analysis

The data were organized with the help of the *Interface de R pour les Analyses Multidimensionnelles de Textes et de Questionnaires* (IRAMUTEQ) software, which provides subsidies to assist a textual analysis, with free access, which is anchored in the R software and the python language to develop diverse textual statistical analyses^(13)^. For content analysis, the speeches formed a single *corpus*, using the Descending Hierarchical Classification (DHC), in which text segments (TS) were organized and classified based on their respective vocabularies and established according to the frequency of their reduced forms (lemmatized words), thus obtaining the TS classes (elementary context unit classes). Soon after, the program helped organize the data in a DHC dendrogram that illustrates the relationships between the classes^(13)^. Based on the elementary context unit classes and dendrogram, an interpretative content analysis was performed by the researcher to name the classes of content grouped by the software. To analyze the speeches, the theoretical methodological framework of manifest content analysis was used, which aims to perform a more critical description at a deeper level of information, taking into account the frequency with which certain characteristics of the content appear and the manifest meanings of statements analyzed^(14)^. In this type of analysis, the researcher reviews the narrative data content, looking for particular words or themes that have been previously typified^(15)^.

### Ethical Aspects

The research followed the ethical and legal precepts regarding Resolution 466/12 and its complementary resolutions, under Opinion 5,069,193. The study guaranteed participant anonymity, who were identified by the codename “Interview”, followed by the order number of the interviews.

## RESULTS

Nine mothers of children with CZVS, aged between 24 and 42 years, took part in the study. The majority were married and mothers of their second child (n = 06), with a high school education level (n = 05), dedicated full-time to caring for children (n = 08), and with a monthly family income corresponding to the Continuous Benefit Payment (In Portuguese, *Benefício de Prestação Continuada* - BPC) (R$ 1,212.00) (n = 06).

**Figure 1:**
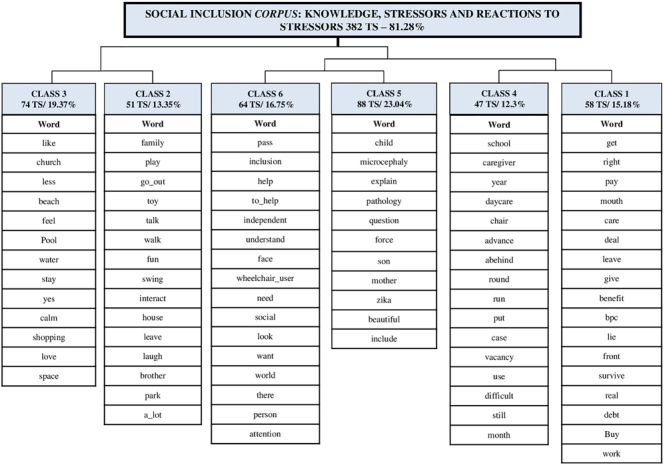
Dendrogram resulting from the Descending Hierarchical Classification of the Social inclusion *corpus*: knowledge, stressors and reactions to stressors, formed by the textual material obtained through the interviews related to the Investigation. Recife, PE, Brazil, 2021.

Source: prepared by the author, 2021.

The interview data generated the text *corpus* called “Social inclusion *corpus*: knowledge, stressors and reactions to stressors”, analyzed from CHD, divided into 470 TS, relating 2,051 words that occurred 16,646 times. The CHD retained 81.28% of the total use of the *corpus*, generating six classes. As shown in Figure 1, first, the *corpus* was divided into two *subcorpora*, the left and the right. The left *subcorpus* gave rise to classes 3 and 2. In a second stage, the right *subcorpus* underwent two further subdivisions, giving rise to classes 5 and 6, as opposed to classes 4 and 1, which resulted in the dendrogram shown, in which it is possible to visualize the results of the association of pre-determined words to the object, such as knowledge, weaknesses and strengths of families on social inclusion in light of Betty Neuman’s theory, which were considered to have significance and high statistical significance regarding the frequency with which they appeared in the text, with a p-value <0.0001, after applying the chi-square test by the software.

After analyzing the dendrogram and reading STs for each class, a strong similarity was observed in the reports between classes 1 and 4, 2 and 3, and 5 and 6, and these were grouped into three categories: Category 1 - Activities outside the home: stressors and reactions to stressors (classes 1 and 4), with 27.5% of TS; Category 2 - Difficulties in adaptation: stressors and reactions to stressors (classes 2 and 3), with 32.7% of TS; Category 3 - Knowledge about rights and inclusion: stressors and reactions to stressors (classes 5 and 6), with 39.8% of TS. Each category, even addressing a specific theme, has, in its vocabulary and TS, thematic strands that contemplate both stress factors (weaknesses/challenges that affect the lines of resistance and defense), as well as reactions that constitute protective elements of the defense lines (strengths/overcoming from the negentropic response) of the family system.

### Category 1 – Activities Outside the Home: Stressors and Strengths

This category, composed of classes 1 and 4, accounted for 27.5% of TS. It refers to issues related to the dynamics experienced by family members regarding the demand for specific care due to the child’s health condition and the performance of various activities outside the home in consultations and therapies, and educational and leisure activities.

This chain of events points to the occurrence of interpersonal and intrapersonal stressors. Attitudes external to the family environment, revealed in barriers in the educational system, weaken family members due to the overload generated in caregivers.

*I have to talk to so-and-so, so-and-so has to talk to so-and-so, and then it just makes things difficult, putting up barriers at school and daycare [...], if there is a vacancy, you still have to run after a caregiver who may not even exist, who may not take care of your child properly, you know, we feel frustrated [...]. (I01)*

*School and daycare, we don’t go, because I don’t trust them, we want to open our mouths and talk, but we hold back and don’t say much, because we feel a little rejected, I don’t know, unprotected. (I04)*

Factors such as lack of support and lack of safety when using public transportation highlight the weaknesses of the family system, causing it to be confined to its home. In this process, families face difficulties in terms of travel, preferring to stay indoors instead of carrying out daily leisure and leisure activities, leading them to social distancing, as we can see in the reports.

*We prefer to stay at home, because of transportation. We don’t go anywhere because there was a bus, but then I go without a seatbelt, I have no support. Sometimes the bus brakes so much that I fall inside the bus, and that’s all for me, for me going out with her. I’m afraid something worse will happen, so I prefer to stay at home [...]. (I06)*

*If I have to expose my son to a hostile place, where there is no proper care for him, I prefer him to stay at home [...]. (I07)*

Although this process of daily challenges faced by families outside the home appears to be arduous and painful, revealing the various types of stressors that affect the family system, it is noted that the statements are endowed with elements that constitute protectors of families’ defense lines, in a movement of reaction to stressors for negentropy (health and well-being), such as maternal self-efficacy in the search to overcome difficulties.

*When I had a problem, I had to make an effort, leave at dawn, leave her with the others so I could go, and there was no way to take her, but I managed, right [...]. (I04)*

*Because I have my tiring side too. There are days when I wake up and look at myself in the mirror and think, I can’t take it anymore, then I look at her like this, and say, I’m going to take care of her [...]. (I06)*

*I go out with him, I take him for a snack, I buy him something he likes, so that, for me, is very important [...]. (I03)*

### Category 2 – Difficulties in Adaptation: Stressors and Strengths

Composed of classes 2 and 3, this category account for 32.7% of TS, and expresses the problems encountered by families in adapting to environments that are not prepared to receive people with disabilities.

The statements reveal a lack of preparation of social facilities to integrate children with CZVS, as well as with other similar health conditions. This factor is a potential generator of stressors for the family, both interpersonal and extrapersonal, evidenced in the following reports:

*In the cinema, that very strong light will cause convulsions [...]. (I01)*

*You go to a square, there’s no way to get on, because few of them have a place, the ramp, it’s all full of holes, it’s very complicated [...]. (I09)*

*It’s difficult, right, because it’s been a short time since she got her wheelchair, but the bus doesn’t have a place to put her wheelchair, it’s not adapted [...]. (I06)*

The difficulty in adapting is generated beyond the barriers in physical equipment. Society also presents itself as a latent interpersonal stressor, through the expression of prejudice, indifference and lack of empathy, weakening the family system’s defense lines.

*What I like least is when someone passes by him, he talks, and the person doesn’t say hello. This empathy that people don’t have, they look at each other differently [...]. (I01)*

*We were in a park, and then a mother came up with a child and asked me why I was there with those other mothers with those children. So, I said that they are my friends and my son has the same pathology as them. Then she said, “Your son is beautiful,” and her special son looks like a monster. So, like, you have no empathy at all, no respect for human beings, right? That made me very sad, right? [...]. (I07)*

*The saddest thing is that not all people are ready to receive different things. They look at me like that and say, “My God!”. For them, it’s as if I have no reason to smile. (I07)*

*When she went to places, she was not welcomed, because people looked at her as if she were an alien [....]. (I09)*

It was observed that, as a positive reaction to people’s lack of empathy, families want to be welcomed in the environments they frequent, and they are happy when this happens. This fact, in turn, is considered a protection of the family system’s defense lines (defense lines ready to face negative situations experienced), as described in the following reports:

*The family is very welcoming. I thought that the family would not accept my daughter, but everything was completely different, because when she was born, my house was visited a lot. The family is welcoming to her and accepts her the way she is [...]. (I04)*

*I feel very welcomed in the church, even she herself. It is a place where she has adapted very well, in the church [...]. (I08)*

The reports make it clear that, despite the difficulties suffered in social environments, the lack of preparation of social equipment, prejudice, among other aspects, being a contributing factor to stress, the family demonstrates the ability to overcome and cope as a positive reaction, through insertion of children in social spaces.

*We go for walks, play with her, interact with her, I think that’s why she’s so smart, she doesn’t stay like a child just sitting there, it’s not because she’s special that she has to stay there, no [...]. (I04)*

*God gives me this resilience that I can get up every day, with faith to overcome difficulties and he will meet my needs and I will succeed [...]. (I07)*

### Category 3 – Knowledge About Rights and Inclusion: Stressors and Strengths

This category is directly related to the need for the family to fully understand their rights, how to claim them and the search for social inclusion. It was composed of classes 5 and 6, which accounted for 39.8% of TS.

When dealing with the issue of social inclusion and the rights of children and families, it was possible to discover a mix of situations: lack of knowledge and/or uncertainty about the rights of the family and children; lack of knowledge about how to act/claim rights; feeling of helplessness in the search for their rights. Reports on this subject are permeated by stressors, both interpersonal and extrapersonal, which weaken the family system. The weaknesses evidenced in the lack of knowledge and/or uncertainty about the rights of the family and children can be seen in the following reports:

*I’ve never been a person who fought much for rights, no, okay? But I think we don’t have half of what we should have, you know [...]. (I01)*

*It’s very difficult to know about rights. I look, I search, I research [...]. (I07)*

Lack of knowledge about how to claim rights generates feelings of abandonment and exclusion as intrapersonal stressors, as we see in the following reports:

*If it were a normal child, no one would forget, but since she has her difficulties, she is forgotten [...]. (I06)*

*What kind of democracy is this? It’s complicated, it’s for him, not for me, it’s for my son, he’s not to blame for being born with the syndrome, it’s his right, and it’s denied [...]. (I07)*

*We don’t have our own rights, and we pay for it [...]. (I09)*

As intrapersonal and extrapersonal stressors, there is also the feeling of impotence reported by the family, when seeking their rights, and the development of a feeling of revolt, when they are not implemented in practice.

*Regarding rights, I feel, well, some things that I see are wrong, I feel revolted, right [...]. (I04)*

*This is my right, I am within my rights, and there are people who do not recognize the rights that we have. (I06)*

*In the face of my rights, I feel very small, because it is very difficult to obtain them. [...]. (I09)*

It was possible to recognize in the report of one participant that, by knowing a right that assists her, she feels stronger to face challenges. It can be said that knowledge about rights is a factor that contributes to the protection of the defense lines of this family system as a potential for overcoming.

*Regarding rights, I feel stronger every day, when I know each right that I learn about that my daughter has, then I feel stronger [...]. (I08)*

Participants expressed their desire to learn more about the rights of their children and families, to consolidate family ties, as another protective factor of the family system.

*I would like to know more about her rights, I should know more, right? [...]. (I02)*

*I would like to know more about her rights, about the rights of a special child [...]. (I04)*

*I don’t know all the rights, but I would like to know. I would like to know even more, so I can help her, and inform other people about what can be done [...]. (I09)*

It was possible to demonstrate that the meaning of “inclusion” for families is still unknown.

*I don’t even know how to explain to you where this social inclusion thing is going, but I’ve heard about it but I don’t understand it [...]. (I02)*

For other participants, the meaning of inclusion refers to acceptance, and the way their child is perceived by society reflects the “non-inclusion” experienced in everyday life.

*For me, social inclusion is when my son arrives somewhere and he is not the center of attention [...]. (I01)*

*For me, social inclusion is like a village, everyone together, everyone supporting. If it were like that, my daughter would not suffer so much bullying today [...]. (I06)*

*Social inclusion is when you welcome and accept people with their limitations. If people were respectful, it would help a lot [...]. (I07)*

*For me, social inclusion is when you can go out with your son and have access to any place you want. Having decent access, like any other person, that is social inclusion [...]. (I09)*

## DISCUSSION

In this study, the primary caregiver of a child with CZVS is shown to be a young, married mother with more than one child and fully dedicated to care. This fact is also found in similar studies^(16–18)^, such as a study carried out with families of children with microcephaly that found a majority profile of female caregivers who were children’s mothers, aged between 17 and 42 years, in a stable relationship, with other children and who did not perform any other function other than that of caregiver^(17)^.

The burden of abandoning employment is recurrent among mothers who take on all the care, as they cannot count on an efficient support network^(19)^. Despite BPC, the financial situation of families remains compromised, as the costs of living and care exceed the value of the benefit^(17,19)^.

The healthcare process for these children is fraught with difficulties due to caregivers’ overload, due to travel to appointments and therapies^(20)^. This fact reflects the importance of strengthening the SUS regarding universal access to healthcare for families affected by CZVS, since most of them depend on the public system^(19,20)^. Betty Neuman’s Systems Model Theory is relevant in this regard, addressing how interpersonal and extrapersonal stressors negatively affect the family system, potentially leading to illness^(8)^. Betty Neuman’s Systems Model Theory addresses this dynamism caused by the invasion of stressors that alter the energy levels of the basic structure of the system and, consequently, can be disease-causing^(8)^.

The correlation between this theory and families of children with CZVS was described in a study^(4)^ that highlighted components that strengthen the defense lines of the family system. By examining the family dynamics of a child with CZVS from the perspective of Betty Neuman’s theory, health professionals are able to foster the appreciation of family ties and the recognition of demands by identifying the strengths inherent in the system’s defense lines. This approach aims to develop educational interventions that promote family autonomy, oriented towards planning holistic and comprehensive care^(4,10)^.

Families of children with CZVS associate their quality of life and well-being with the provision of good services by organizations and institutions maintained by the government^(21)^, whether in health, education, transportation, social security and other forms of social assistance^(22)^.

The lack of support from institutions, which is an extra-personal stressor, generates a lack of trust and social withdrawal. The lack of seat belts and wheelchair access adaptations was highlighted as a requirement that generates insecurity when using the bus, when it is evident that the standards required for private transportation are not ensured in relation to public transportation. One of the greatest issues faced by families is dealing with the need for mobility and obtaining co-participation in care^(4–23)^.

Even though adversities are numerous and are part of families’ daily lives, the act of overcoming them is present in the participants’ discourse, who insist on seeking the best for their children, through the commitment to safeguard their safety and protect them from an environment that is sometimes considered hostile. For Neuman, the family system’s defense lines, particularly the flexible line, cushion the impact of stressors, playing the role of a shield for the system’s stability^(8)^.

Accessibility is a citizen’s right and is directly related to the right to leisure, happiness and freedom of movement. Physical, architectural and attitudinal barriers that hinder or prevent people with disabilities from enjoying life in society must be eliminated^(24)^. The problems encountered by families in adapting children to environments outside the home demonstrate that the environments are not prepared to receive people with disabilities, and are characterized as exclusionary spaces to ensure that mothers have the possibility of developing the necessary care for their children with CZVS.

Exposure of children outside the home triggers stressors that affect the system’s protective defenses. Families suffer prejudice, indifference, and a lack of empathy from society. The psychosocial fragility of the ZIKV epidemic generates disrespect, embarrassment, and violence toward children and their families, calling into question the responsibility that exists in society and in the government, as provided for by law, such as the Statute of Children and Adolescents, which is responsible for safeguarding children’s rights^(25)^.

The family, as a support network, formed by a set of systems and significant people, between relationships received and perceived by individuals^(25)^, allows the construction of horizontal relationships in social spaces, enabling the leading role of families in the care of their members, in addition to being a guarantee for the validity of the much desired acceptance and respect in the face of discrimination and intolerance due to children’s health condition^(26,27)^.

There is a lack of knowledge about the rights that families have, as well as the ways to claim them, to promote social inclusion, because despite legal advances on the educational and social rights of people with disabilities in Brazil, families do not feel supported by the State, making it necessary to frequently resort to the Judiciary to obtain validity of these rights^(28)^.

The feeling of helplessness reported by mothers in the fight to assert their rights stands out as a stressor for the family system. The challenges of congenital microcephaly have shown that, at the height of the health crisis, the rights of families were not respected, demonstrating the omission on the part of public agents that generated a large number of lawsuits, including the participation of the Federal Public Prosecutor’s Office in a lawsuit in the Federal Court to guarantee medical treatment and comprehensive care for children with microcephaly^(29)^.

The story of a mother who claims that knowledge about a right makes her stronger to face challenges shows the human capacity to embrace opportunities as a way of improving oneself in a crisis situation^(18)^. There is a tendency among families of children with disabilities to counter weaknesses through possibilities of developing resilience and overcoming crises^(30,31)^, with nursing being responsible, in Betty Neuman’s theory, for carrying out interventions in the system that can promote health and balance^(8)^.

Betty Neuman advocates as a proposal for intervention in the system the development of actions that generate social and emotional support to obtain a strengthened system, with integral defense lines. A balanced family system is strengthened from the positive effects arising from interactions with the environment^(8)^.

## STUDY LIMITATIONS

Considering that the study was developed in a reference unit for therapeutic monitoring of children with CZVS in the countryside of the state of Pernambuco, Brazil, it is impossible to generalize the results to all families in the context of CZVS. However, this study can be replicated in other groups of families of children with CZVS who experience dynamics similar to those of the participants in this research, making it possible to encourage discussion of stressors and their respective reactions to generate improvements in accessibility conditions and, consequently, in the process of social inclusion.

## CONTRIBUTIONS TO NURSING AND HEALTH

The results of this research contribute to expanding the knowledge of nursing and other professionals in the areas of health, law, social assistance and government, especially with regard to care and accessibility, with the formation of effective public policies, built based on the specific needs of children with CZVS and their families, taking into account the details of the daily lives of this population due to health conditions, housing, transportation, and access to legal goods and services. It is expected that the results presented will support new scientific productions that allow for real investigation of the need and effective guidance for solving problems.

## CONCLUSION

The empirical data revealed that the knowledge and experiences of families of children with SCZ regarding social inclusion in light of Betty Neuman permeate the exhaustive dynamics of the demand for care, the need to provide children with experiences of social interaction, the attempt to adapt to unsuitable environments, the suffering caused by prejudice and discrimination, the struggle to seek validity of their rights and the search for social inclusion, factors that permeate the daily lives of families.

Betty Neuman’s theory allowed for the refined analysis of a system constantly affected by stressors, in which a reaction movement is observed to maintain the system in balance. Furthermore, it was possible to reflect that knowledge of the stressor is evident as the main strategy for organizing the system and using the available energies in a reverse dynamic of strength and overcoming.

## References

[1] Iani FCM, Giovanetti M, Fonseca V, Souza WM, Adelino TER, Xavier J (2021). Epidemiology and evolution of Zika virus in Minas Gerais, Southeast Brazil. Infect Genet Evol.

[2] Teixeira GA, Silva AN, Miranda LSMV, Silva MPM, Cavalcante EFO, Enders BC (2021). Theoretical care model for children with congenital Zika virus syndrome in the family context. Rev Lat Am Enfermagem.

[3] Souza MPA, da Natividade MS, Werneck GL, dos Santos DN (2022). Congenital Zika syndrome and living conditions in the largest city of northeastern Brazil. BMC Public Health.

[4] Lima LHSS, Monteiro EMLM, Coriolano MWL, Linhares FMP, Cavalcanti AMTS (2020). Family fortresses in Zika Congenital Syndrome according to Betty Neuman. Rev Bras Enferm.

[5] Morris M, Brito A, Malta M, Jacques I, Rocha G, Novaes R (2022). Experiences of women raising children with congenital Zika syndrome along a trajectory of prevention, care and support in Brazil. Glob Public Health.

[6] Mendes AG, Campos DS, Silva LB, Moreira MEL, Arruda LO (2020). Enfrentando uma nova realidade a partir da síndrome congênita do vírus zika: a perspectiva das famílias. Cien Saude Colet.

[7] Cruvinel SP (2023). Inclusão social? De quem e para quem? Humanidades e Tecnologia (FINOM).

[8] Mcewen M, Wills EM (2015). Bases teóricas de enfermagem.

[9] Neuman B, Fawcett J (2011). The Neuman systems model.

[10] Lima LHSS, Cavalcanti AMTS, Linhares FMP, Morais SCRV, Souza KV, Duarte ED, Associação Brasileira de Enfermagem, Associação Brasileira de Obestetrizes e Enfermeiros Obstetras (2018). Proenf Programa de Atualização em Enfermagem: Saúde Materna e Neonatal: Ciclo 10.

[11] Flick U (2009). Desenho da pesquisa qualitativa.

[12] Tong A, Sainsbury P, Craig J (2007). Consolidated criteria for reporting qualitative research (COREQ): a 32-item checklist for interviews and focus groups. Int J Qual Health Care.

[13] Camargo BV, Justo AM (2013). IRAMUTEQ: um software gratuito para análise de dados textuais. Temas Psicol.

[14] Minayo MCS, Deslandes SF, Cruz NO, Gomes R (1994). Pesquisa social: teoria, método e criatividade.

[15] Polit DF, Beck CT, Hungler BP (2004). Fundamentos de pesquisa em enfermagem.

[16] Marinho F, Araújo VEM, Porto DL, Ferreira HL, Coelho MRS, Lecca RCR (2016). Microcefalia no Brasil: prevalência e caracterização dos casos a partir do Sistema de Informações sobre Nascidos Vivos (Sinasc), 2000-2015. Epidemiol Serv Saude.

[17] Menezes MGV, Mendes No JM, Leal CNL, Vasconcelos APL, Aragão HT, Silva NV (2019). Dificuldades e estratégias da família no cuidado da criança portadora de microcefalia: Family difficulties and strategies in children’s care with microcephaly. Rev Enferm Atual In Derme.

[18] Sá SAAG, Galindo CC, Dantas RS, Moura JC (2020). Dinâmica familiar de criança com a síndrome congênita do Zika vírus no Município de Petrolina, Pernambuco, Brasil. Cad Saude Publica.

[19] Freitas PSS, Soares GB, Mocelin HJS, Lacerda LCX, Prado TN, Sales CMM (2019). Síndrome congênita do vírus Zika: perfil sociodemográfico das mães. Rev Panam Salud Publica.

[20] Costa AM (2016). A determinação social da microcefalia/Zika no Brasil. Cad Trab Rede Water-Lat-Gobacit.

[21] Costa PRA, Aragão FBA, Serra JN, Andrade MS, Reis AD, Nascimento MDSB (2021). Quality of life of mothers of children with congenital syndrome affected by Zika virus. Rev Paul Pediatr.

[22] Scott P (2020). Cuidados, mobilidade e poder num contexto de epidemia: relações familiares e espaços de negociação. Mana.

[23] Williams NA, Villachan-Lyra P, Marvin C, Chaves E, Hollist C, Hatton-Bowers H (2021). Anxiety and depression among caregivers of young children with congenital Zika syndrome in Brazil. Disabil Rehabil.

[24] Peiter PC, Pereira RS, Nunes Moreira MC, Nascimento M, Tavares MFL, Franco VC (2020). Zika epidemic and microcephaly in Brazil: challenges for access to health care and promotion in three epidemic areas. PLoS One.

[25] Azevedo CS, Freire IMM, Farias LN (2020). “Aí começou a saga...”: fragilidade psicossocial na epidemia do vírus Zika. Cad Saude Publica.

[26] Mendes AG, Campos DS, Silva LB, Moreira MEL, Arruda LO (2020). Enfrentando uma nova realidade a partir da síndrome congênita do vírus zika: a perspectiva das famílias. Ciênc Saúde Colet.

[27] Bulhões CSG, Almeida AM, Reichert APS, Abreu PD, Dias MD (2020). Oral History of Mothers of Children with Congenital Zika Virus Syndrome. Texto Contexto Enferm.

[28] Calheiros de Sá MR, Pletsch MD (2021). A participação de crianças com a Síndrome Congênita do Zika Vírus: interseções entre o modelo bioecológico e a funcionalidade humana. Prax Educ.

[29] Casemiro ÍP (2021). Todo cuidado do mundo: mulheres e o desafio da microcefalia congênita. Rev Eletron Comun Inf Inov Saúde.

[30] Silva SC, Dessen MA (2014). Family relationships: a perspective of parents, siblings and children with deficiency. Rev Bras Educ Espec.

[31] Juliano MCC, Yunes MAM (2014). Reflexões sobre rede de apoio social como mecanismo de proteção e promoção de resiliência. Ambiente Soc.

